# A reduction in perceived psychological distress over three years is associated with improvements in pain and symptoms in patients with longstanding hip and groin pain - a longitudinal prospective cohort study

**DOI:** 10.1186/s12891-026-10231-2

**Published:** 2026-07-18

**Authors:** Anders Pålsson, Andreas Ivarsson, Eva Ageberg

**Affiliations:** 1 Department of Health Sciences, Faculty of Medicine, Lund University, Lund, Sweden; 2 Centre of Research on Welfare, Health and Sport, Halmstad University, Halmstad, Sweden

**Keywords:** Psychological distress, Hip, Groin, Pain, Longitudinal studies

## Abstract

**Background:**

Previous cross-sectional studies report an association between psychological distress and severity of pain and symptoms in patients with longstanding non-arthritic hip and groin pain (LHGP). However, there is a lack of longitudinal studies exploring changes in psychological distress over time, and whether these changes relate to changes in pain and symptoms in LGHP patients. This study aimed to assess any changes in patient-reported psychological distress in patients with LHGP from the time of referral to orthopedic care to a follow-up three years later. An additional aim was to explore the relationship between changes in psychological distress and changes in patient-reported pain and symptoms from baseline to the three-year follow-up.

**Methods:**

Eighty-one patients who had previously participated in a cross-sectional study were invited for follow-up three years after their referral for orthopedic care due to LHGP. The Kessler Psychological Distress Scale (K-10) was used to assess psychological distress, and the subscales for pain and symptoms in the Copenhagen Hip and Groin Outcome Score (HAGOS) was used to assess pain and symptoms. The Wilcoxon signed-rank test was used for comparisons of K-10 scores from baseline to follow-up, and Spearman’s rank order correlation was used to examine the association between changes in K-10 scores and changes in HAGOS scores.

**Results:**

Forty-five (56%) patients participated in the follow-up. No significant group-level change in the K-10 total score was found from baseline to the three-year follow-up (median change 0.0, IQR − 4.25; 4.25, *p* = 0.792). Significant improvements in the HAGOS subscales for pain (mean change 14.3, 95%CI 7.8;20.8) and symptoms (16.4, 95%CI 8.9;24.0) were reported (*p* < 0.001). A moderate association was observed between changes in the K-10 total score and changes in the HAGOS subscale for pain (r_s_=-0.436, *p* = 0.004) and symptoms (r_s_=-0.431, *p* = 0.005) from baseline to the three-year follow-up.

**Conclusions:**

Despite no significant group-level change in psychological distress, reductions in psychological distress were moderately associated with improvements in pain and symptom scores. The relationship between psychological distress and hip and groin pain and symptoms highlights the importance of considering both physical and psychological factors in patient management.

**Supplementary Information:**

The online version contains supplementary material available at 10.1186/s12891-026-10231-2.

## Background

Patients with longstanding hip and groin pain (LHGP) experience reduced physical function and lower quality of life compared to individuals without chronic pain [1–4]. Commonly reported symptoms in this patient group include pain, stiffness, and discomfort in the hip during daily activities as well as during sports and recreational physical activities [1–4].

Pain and symptoms in patients with longstanding hip and groin pain are likely influenced by a complex interaction of biological, psychological, and social factors. Biological factors, such as hip joint morphology, chondral damage, labral tears, and tendinopathies, may contribute to pain and symptoms in some individuals [5, 6]. However, these factors alone do not fully explain the variability in pain severity or symptom burden between patients. Psychological factors, including symptoms of anxiety and depression, may also influence the perception and experience of pain and contribute to differences in pain and symptom severity among patients with longstanding hip and groin pain [7, 8].

Previous cross-sectional studies have demonstrated associations between psychological distress and the severity of pain and symptoms in patients with LHGP [9–12]. Furthermore, Fischer et al. [13] showed that psychological factors such as depression and somatization are significant risk contributors for worse hip function and activity levels following periacetabular osteotomy. Similarly, Gudmundsson et al. [14] found that improvements in hip function over six months were closely linked to reductions in pain catastrophizing, anxiety, and depression after treatment, highlighting a bidirectional relationship between physical and psychological recovery in patients with hip pathology. However, there remains a lack of long-term longitudinal studies investigating whether changes in psychological distress relate to pain and symptom severity in patients with LHGP. Such knowledge could provide valuable insights into prognostic factors and support the development of integrated management strategies, including targeted psychological interventions, to optimize long-term outcomes for patients with LHGP.

Therefore, the present study aimed to assess changes in patient-reported psychological distress among individuals with LHGP from the time of referral to orthopedic care to a three-year follow-up. A secondary aim was to explore the relationship between changes in psychological distress and changes in patient-reported pain and symptoms over the same period.

## Methods

### Study design and ethics

This prospective longitudinal exploratory study adhered to the “Strengthening The Reporting of Observational Studies in Epidemiology” (STROBE statement) [15] and was registered on Clinical Trials.gov (ClinicalTrials.gov identifier: NCT03490071). The study was conducted in accordance with the Declaration of Helsinki and the Swedish Ethical Review Authority approved the study (Dnr:2014/12 and 2017/671). The participants signed an informed consent form prior to data collection.

### Participants

Eighty-one patients who had previously participated in a cross-sectional study [4] were invited for follow-up three years after their referral for orthopedic care due to LHGP. After inclusion at baseline, patients were categorized as having either hip joint–related pain or non–hip joint–related pain, according to the diagnostic criteria (symptoms, clinical signs, imaging findings, and response to intra-articular injection) described by Griffin et al. [5].

### Data collection

Participant characteristics and patient-reported outcome measures were collected via the Sunet Survey (Artologic©, Sweden) hosted by Lund University at baseline. At the three-year follow-up, REDCap (Research Electronic Data Capture, version 9.3.1) hosted by Lund University was used.

### Instruments

#### Kessler psychological distress scale

The Kessler psychological distress scale (K-10) is a 10-item questionnaire that measures psychological distress, i.e.,symptoms of depression and anxiety. Patients rate each item based on their experiences over the past four weeks. Each item is rated on a best-to-worst scale ranging from 1 (none of the time) to 5 (all of the time). The total score is calculated by summing the scores of all items (range, 10–50), with higher scores indicating higher levels of psychological distress [16]. The K-10 is a widely used screening instrument for non-specific psychological distress with established validity in general and clinical populations [17]. However, it is primarily designed for screening purposes and clear thresholds for minimal detectable or clinically important change are not well established.

#### Copenhagen hip and groin outcome score

The Copenhagen Hip and Groin Outcome Score (HAGOS) is a hip-specific questionnaire consisting of six subscales: pain, symptoms, activities of daily living, physical function in sport and recreation, participation in physical activity, and quality of life. Patients rate each subscale based on their experiences over the past week. Each subscale is scored from 0 to 100, where 0 indicates extreme problems and 100 indicates no problems. The HAGOS has been shown to be a reliable and valid instrument for assessing LHGP in young to middle-aged adults [18]. In this study, the subscales pain and symptoms were used. Published minimal important change (MIC) values for the HAGOS pain and symptom subscales are 10 and 6 points, respectively [19].

### Statistics

Data were analyzed using SPSS Statistics version 26 (IBM, Armonk, USA). The original cross-sectional study [4] did not include a predefined sample size calculation because of its exploratory design, and the current study therefore adopted a similar exploratory approach. Non-parametric methods were applied to the K-10 scores due to the ordinal nature of the data. The Wilcoxon signed-rank test was used to compare K-10 scores from baseline to follow-up. Spearman’s rank-order correlation was employed to explore the association between changes (follow-up score minus baseline score) in K-10 and HAGOS scores over the three-year period. Correlation coefficients were interpreted as follows: 0.1 (small), 0.3 (moderate), 0.5 (large), 0.7 (very large), and 0.9 (extremely large) [20]. For drop-out analysis, a t-test was used to compare K-10 scores and HAGOS scores for pain and symptoms in participants and non-participants.

## Results

Forty-five (56%) patients participated in the follow-up. Two patients had missing HAGOS data at baseline, one patient had missing K-10 data at baseline, and one patient had missing K-10 data at follow-up. Drop-out analysis showed no differences (*p*≥0.098), (effect size *d*≤0.407) in K-10, or in HAGOS subscale for symptoms and pain between patients who participated in the follow-up and those who did not (Appendix, Table A). Patients characteristics are presented in Table 1.

**Table 1:** Patient characteristics, diagnostic categorization, HAGOS subscale score and K-10 score at baseline and at three-year follow-up, and time from baseline to follow-up. (n=45 unless otherwise stated)

	Baseline	Three-year follow-up
Sex, n (%) female	22 (48)	n.a
Age, years, mean(SD)	36 (9)	n.a
BMI, mean (SD)	25.19 (3.82)	n.a
Diagnostic categorization, n (%)		
Hip joint -related pain	22 (49)	n.a
Non hip joint-related pain	18 (40)	n.a
Unknown*	5 (11)	n.a
HAGOS subscale score, mean (SD)		
Pain	60.5 (16.2)**	71.3 (16.4)
Symptom	57.8 (15.2)**	75.9 (16.9
K-10 total score, median (IQR), n=44***	19.0 (15.0-25.0)***	19.5(17.0-24.8)****
Time from baseline to follow-up, months, mean (SD)	38.6 (3.0)	

*SD *Standard deviation, *IQR* Interquartile range, *n.a *Not applicable

*Five patients had missing data from the diagnostic injection

**Two patients had missing HAGOS data at baseline

***One patient had missing K-10 data at baseline

****One patient had missing K-10 data at follow-up

**Fig. 1:**
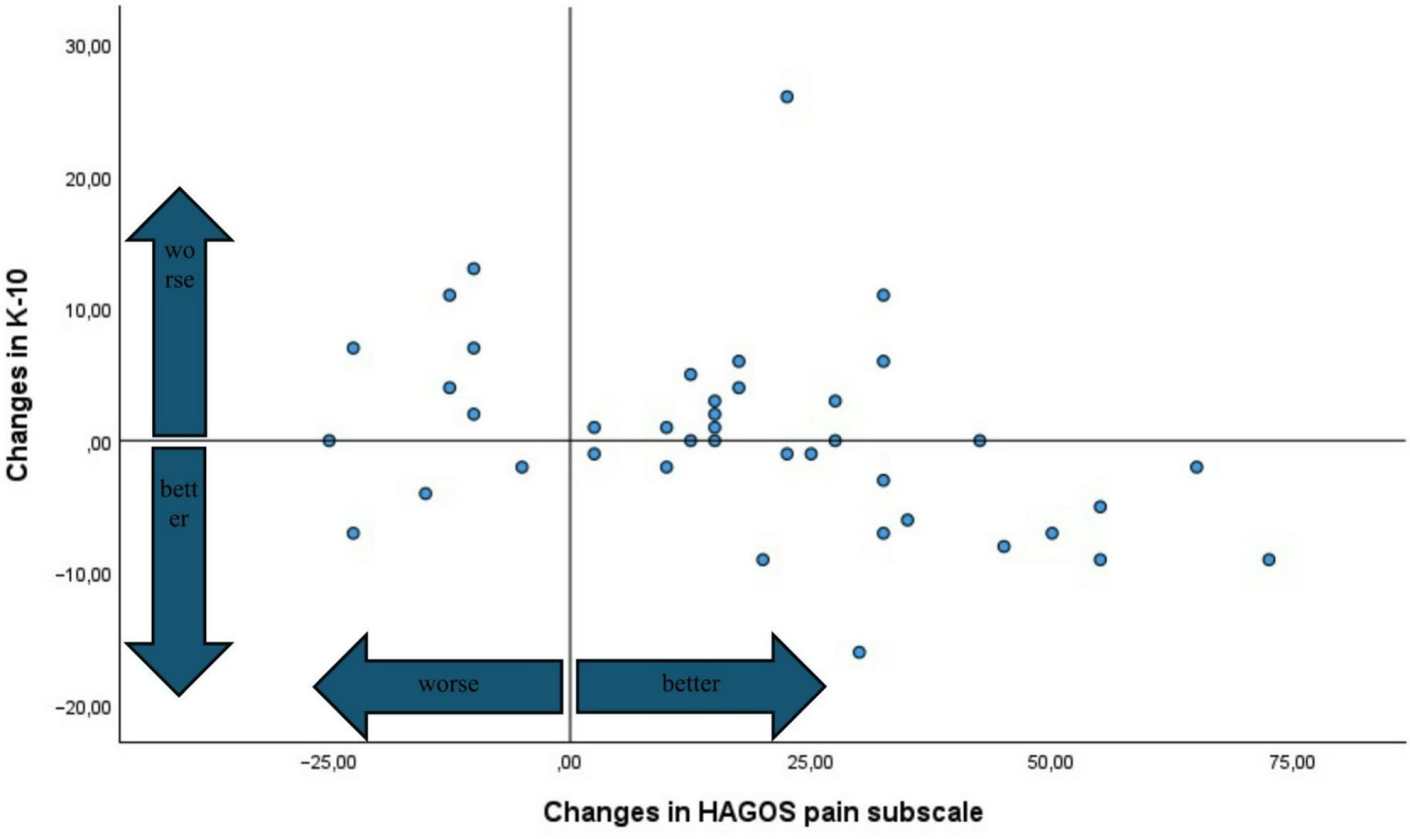
Scatter plot of changes in K-10 and HAGOS pain subscale (*n* = 41)

**Fig. 2:**
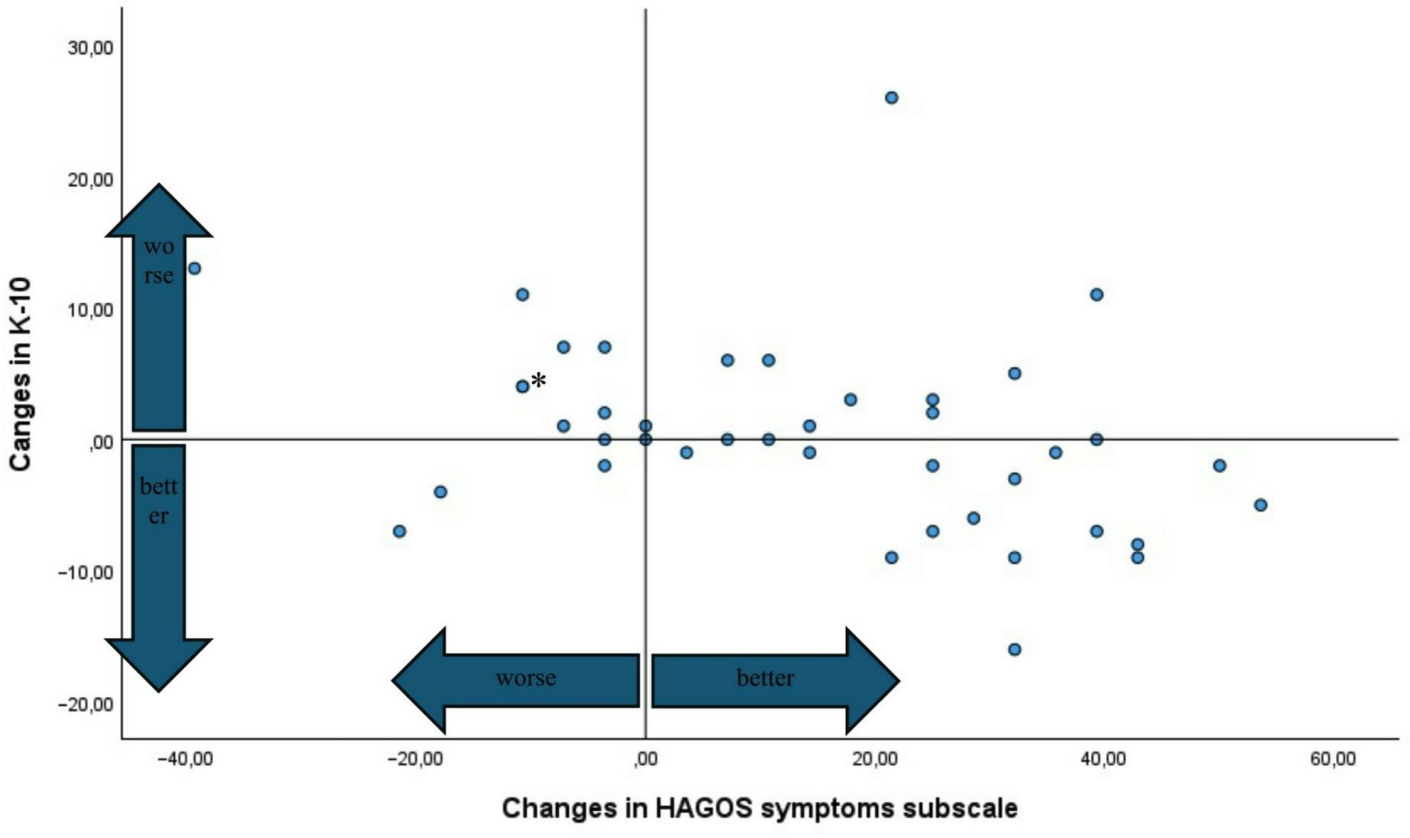
Scatter plot of changes in K-10 and HAGOS symptom subscale (*n* = 41). *= Two participants with identical change scores in both the HAGOS symptom subscale and the K-10, and therefore appearing as a single point

No significant group-level change in the K-10 total score was found from baseline to the three-year follow-up (median change 0.0, IQR − 4.25; 4.25, *p* = 0.792). A moderate association was observed between decreases in K-10 total score (indicating reduced psychological distress) and increases in HAGOS pain (r_s_ = − 0.436, *p* = 0.004; Fig. 1) and symptom scores (r_s_ = − 0.431, *p* = 0.005; Fig. 2) (indicating improved outcomes) from baseline to the three-year follow-up.

## Discussion

In this longitudinal follow-up of patients with LHGP, no significant group-level change in psychological distress was observed over three years. However, individual reductions in psychological distress were moderately associated with improvements in pain and symptom scores.

No significant group-level change in psychological distress was observed over the three-year follow-up. About 50% of the participants reported relatively low levels of psychological distress at baseline (median K-10 score 19), corresponding to low to moderate psychological distress [12]. Consequently, there may have been limited scope for further improvement in psychological distress over time. In addition, changes in K-10 scores were generally modest, with most participants showing changes within approximately ± 8 points. Given that minimal detectable change (MDC) and minimal clinically important difference (MCID) values for the K-10 have not been established, it is difficult to determine whether these individual changes represent clinically meaningful changes in psychological distress or normal fluctuations over time. This may partly explain why similar proportions of participants showed improvements and worsening in psychological distress, despite no significant group-level change. Consequently, the observed associations between changes in psychological distress and changes in pain and symptoms should be interpreted with caution.

Because few previous studies have examined long-term changes in psychological distress in patients with longstanding hip and groin pain, meaningful comparisons are challenging. Gudmundsson et al. [15] reported significant improvements in symptoms of anxiety and depression six months after treatment for hip pathology. The lower baseline level of psychological distress in the present cohort, together with differences in study population, outcome measures, treatment exposure, and follow-up duration, may partly explain the differing findings.

The moderate correlations between changes in K-10 and HAGOS subscales for pain and symptoms are consistent with a bidirectional relationship between psychological and physical factors in musculoskeletal pain conditions [14, 21, 22]. Gudmundsson et al. [14], reported that improvements in function following hip pathology treatment are related to reduced anxiety, depression, and pain catastrophizing. Similarly, Fischer et al. [13] reported that psychological factors are important predictors of hip function after hip surgery. The present study extends these findings by demonstrating that such associations persist even over a longer follow-up period and across a heterogeneous group of patients with LHGP. The findings from both the present and previous longitudinal studies [13, 14] highlight the importance of integrating a biopsychosocial approach in the management of patients with LHGP.

### Strenghts and limitations

To our knowledge, our study is the first to assess psychological distress over time in a long-term perspective in patients with LHGP. Several limitations should be acknowledged. The relatively small sample size (*n* = 41) limits statistical power and reduces the ability to detect smaller changes or more subtle associations. Also, the follow-up rate of 56% introduces a potential risk of attrition bias. Although no clinically relevant differences were observed between participants and non-participants at baseline, it cannot be excluded that the follow-up rate may affect the representativeness of the sample and limit the generalisability of the findings.

The K-10 measures general psychological distress without distinguishing between anxiety and depressive symptoms, which may have different relationships with pain and function. Furthermore, the K-10’s four-week recall period may not reflect longer-term psychological changes across the three-year interval. Also, K-10 is primarily designed as a screening tool for psychological distress and may be less sensitive to detecting small but clinically meaningful changes over time. Moreover, minimal detectable change (MDC) or minimal clinically important difference (MCID) values for the K-10 are not well established, making it difficult to distinguish true change from measurement variability. Consequently, the observed associations between changes in psychological distress and changes in pain and symptoms should be interpreted with caution.

Given the exploratory design, and due to the small sample size and the ordinal nature of the data, we chose a simple non-parametric approach ta assess the assosciation between changes in HAGOS scores and K10 scores. Therefore, the study did not control for potential confounding variables such as diagnostic categorisation (hip joint–related vs. non–hip joint–related pain), treatment exposure, physical activity, or life events that could have influenced outcomes.

Finally, the observational design prevents causal interpretations. While reduced psychological distress was associated with decreased pain and symptoms, it cannot be determined whether one caused the other.

### Clinical implications

The observed associations between changes in psychological distress and changes in pain and symptoms support the relevance of considering psychological factors in patients with longstanding hip and groin pain. However, given the observational design and the lack of significant change in psychological distress at the group level, these findings should be interpreted with caution and do not imply causality. The results may suggest that psychological distress and physical symptoms are interrelated over time, potentially in a bidirectional manner. As such, a biopsychosocial perspective may be relevant when assessing and managing patients with longstanding hip and groin pain. Future research is needed to determine whether targeting psychological factors leads to improved clinical outcomes.

## Conclusion

Although no significant group-level change in psychological distress was observed over three years, reductions in psychological distress were moderately associated with improvements in pain and symptoms. These findings highlight the interplay between psychological and physical factors in LHGP and emphasize the need to consider psychological factors as a component of patient management and rehabilitation strategies.

## Supplementary Information

Supplementary Material 1.

## Data Availability

The data used in this study contain sensitive information about the study participants who did not provide consent for public data sharing. The current approval by the Swedish Ethical Review Authority approved the study (Dnr:2014/12 and 2017/671) does not include data sharing. A minimal data set could be shared on request from a qualified academic investigator for the sole purpose of replicating the present study, provided the data transfer is in agreement with EU legislation on the General Data Protection Regulation and approval by the Swedish Ethical Review Authority.Contact information: Department of Health Sciences, Lund University, Box 117, 221 00 Lund, Sweden. Contact address: [DHSdataaccess@med.lu.se](mailto: DHSdataaccess@med.lu.se) ; Principal investigator: Eva Ageberg Eva Ageberg, Department of Health Sciences, Faculty of Medicine, Lund University, PO Box 117, SE-221 00 Lund, Sweden; email: [eva.ageberg@med.lu.se](mailto: eva.ageberg@med.lu.se) ; Swedish Ethical Review Authority, Box 2110, 75 002 Uppsala, Sweden. Phone: +46 10 475 08 00.

## Abbreviations

LHGP: Longstanding hip and groin pain
K-10: Kessler Psychological Distress Scale
HAGOS: Copenhagen Hip and Groin Outcome Score
IQR: Interquartile range
SD: Standard deviation
