# Supplementary material for: A reduction in perceived psychological distress over three years is associated with improvements in pain and symptoms in patients with longstanding hip and groin pain - a longitudinal prospective cohort study
Source: BMC Musculoskelet Disord. 2026 Jul 18;27:613. doi: 10.1186/s12891-026-10231-2 (PMC13379962; doi:10.1186/s12891-026-10231-2)
Supplement: Supplementary file 1 — Supplementary Material 1. [file 12891_2026_10231_MOESM1_ESM.docx]

# Appendix

Table A. Patient characteristics, HAGOS subscale score and K-10 score at baseline for participants (n=45) and non-participants (loss to follow-up) (n=36).

|  | Participants (n=45) | Non-participants (n=36) |
| --- | --- | --- |
| Sex, n (%) female | 22 (48) | 18 (50) |
| Age, years, mean(SD) | 36 (9) | 35 (9) |
| BMI, mean (SD) | 25.19 (3.82) | 24.36 (4.04) |
| HAGOS subscale score, mean (SD)   - Pain - Symptom | 60.5 (16.2)  57.8 (15.2) | 53.6 (17.7)  54.9 (15.6) |
| K-10 total score, median (IQR) | 19.0 (15.0-25.0) | 21.0 (16.0-26.0) |
